# Harnessing Artificial Intelligence (AI) in Anaesthesiology: Enhancing Patient Outcomes and Clinical Efficiency

**DOI:** 10.7759/cureus.73383

**Published:** 2024-11-10

**Authors:** Arnesh Shukla, Ayesha Salma, Dev Patel, Jabez David John, Reshmitha Kantamneni, Tirath Patel, Ketan Kantamaneni

**Affiliations:** 1 Psychiatry, St. Martinus University, Foster City, USA; 2 Internal Medicine, Shadan Institute of Medical Sciences, Hyderabad, IND; 3 Internal Medicine, Lokmanya Tilak Municipal Medical College and General Hospital, Mumbai, IND; 4 Surgery, California Institute of Behavioral Neurosciences and Psychology, Fairfield, USA; 5 Medicine, Rangaraya Medical College, Kakinada, IND; 6 Medicine, American University of Antigua, St. John, ATG; 7 Trauma and Orthopaedics, East Kent University Hospitals NHS Foundation Trust, Ashford, GBR

**Keywords:** ai, anesthesiology, artificial intelligence (ai), clinical efficiency, patient outcomes

## Abstract

The rapid rise and potential of artificial intelligence (AI) have created growing excitement and much debate on its potential to bring transformative changes across entire industries, including the medical industry. This systematic review aims to investigate the advancements in the AI industry and its potential implementation, specifically in the field of anaesthesiology. AI has already been integrated into different areas of medicine, including diagnostic uses in radiology and pathology and therapeutic and interventional uses in cardiology and surgery. In the field of anaesthesiology, AI has made significant progress. Potential applications include personalised drug dosing, real-time monitoring of vital signs, automated anaesthesia delivery systems, and predictive analytics for adverse events. As AI technologies continue to advance and become more prevalent in medicine, clinicians across all specialities need to understand these technologies and how they can be utilised to provide safer and more efficient care. With the rapid evolution of AI and the introduction of new concepts such as machine learning (ML), deep learning (DL), and neural networks, the field of anaesthesiology is set to undergo transformative changes. In this systematic review, we examine the existing literature to explore the current state of AI in the field of anaesthesiology, along with a prospective look at potential applications in the future. Along with its various applications, we will also discuss its limitations and flaws. As the field progresses, it is crucial to thoughtfully examine the ethical aspects of using AI in anaesthesia and ensure these technologies are applied responsibly and transparently.

## Introduction and background

In the ever-evolving realm of modern healthcare, artificial intelligence (AI) is set to revolutionise the delivery of medical services, offering unprecedented opportunities to enhance patient care and drive innovation in diagnostics and treatment. According to a report by Market Research Future, the global AI in the healthcare market is expected to reach a value of over $51 billion by 2027, with a compound annual growth rate of approximately 51% from 2020 to 2027 [1]. This report highlights AI's expected growth, adoption, and implementation across all healthcare segments. As AI evolves and advances at a rapid pace, it is crucial for physicians and hospitals to responsibly integrate and utilise its capabilities to optimise the delivery of healthcare services.

AI is a field of computer science that identifies and predicts patterns in large datasets [2]. AI can be further divided into subfields that specialise in different approaches to learning and problem-solving. Machine learning (ML) is a subset of AI that focuses on enabling computers to learn from data and improve over time without being explicitly programmed. Instead of relying on predefined rules, ML algorithms use patterns and inference to make decisions and predictions. On the other hand, deep learning (DL) is a more advanced form of ML inspired by the structure and function of the human brain's neural networks. DL algorithms, known as artificial neural networks, consist of multiple layers of interconnected nodes (or neurons) that process and analyse data hierarchically [3]. These networks handle large, complex datasets and are particularly effective in image and speech recognition tasks. DL has revolutionised fields such as computer vision and natural language processing, enabling machines to understand and interpret information with human-like accuracy. Reinforcement learning refers to the process by which an algorithm is asked to attempt a particular task (e.g., deliver inhalational anaesthesia to a patient or drive a car) and to learn from its subsequent mistakes and successes [4]. Through reinforcement learning, the AI system aims to maximise cumulative reward over time by learning optimal strategies through trial and error, and understanding how these various subfields of AI work is essential to implementing these systems in the field of healthcare effectively and to recognising the limitations of the current state of AI.

Although the advantages and uses of integrating AI in healthcare are well documented, a careful look at the current limitations of AI is necessary to fully understand both its potential and drawbacks, as well as to address any challenges that may arise. Some of these limitations include the need for high-quality data, the potential for bias and error, and the ethical and regulatory considerations that must be addressed [5]. Some of the limitations of classical AI were particularly apparent in attempts to produce programs capable of playing Go. Even the best classical AI approaches to Go seemed unable to accomplish anything better than amateur play; however, in March 2016, a computer program, AlphaGo (DeepMind Technologies, London, UK), defeated a human player of the highest professional calibre, the world number two Lee Sedol, in a head-to-head five-game series of Go. This was the first time a computer program had beaten a player of that skill level without handicaps [6]. As AI continues to evolve, ongoing research and development may help to mitigate these limitations, leading to more effective applications in healthcare.

**Figure 1:**
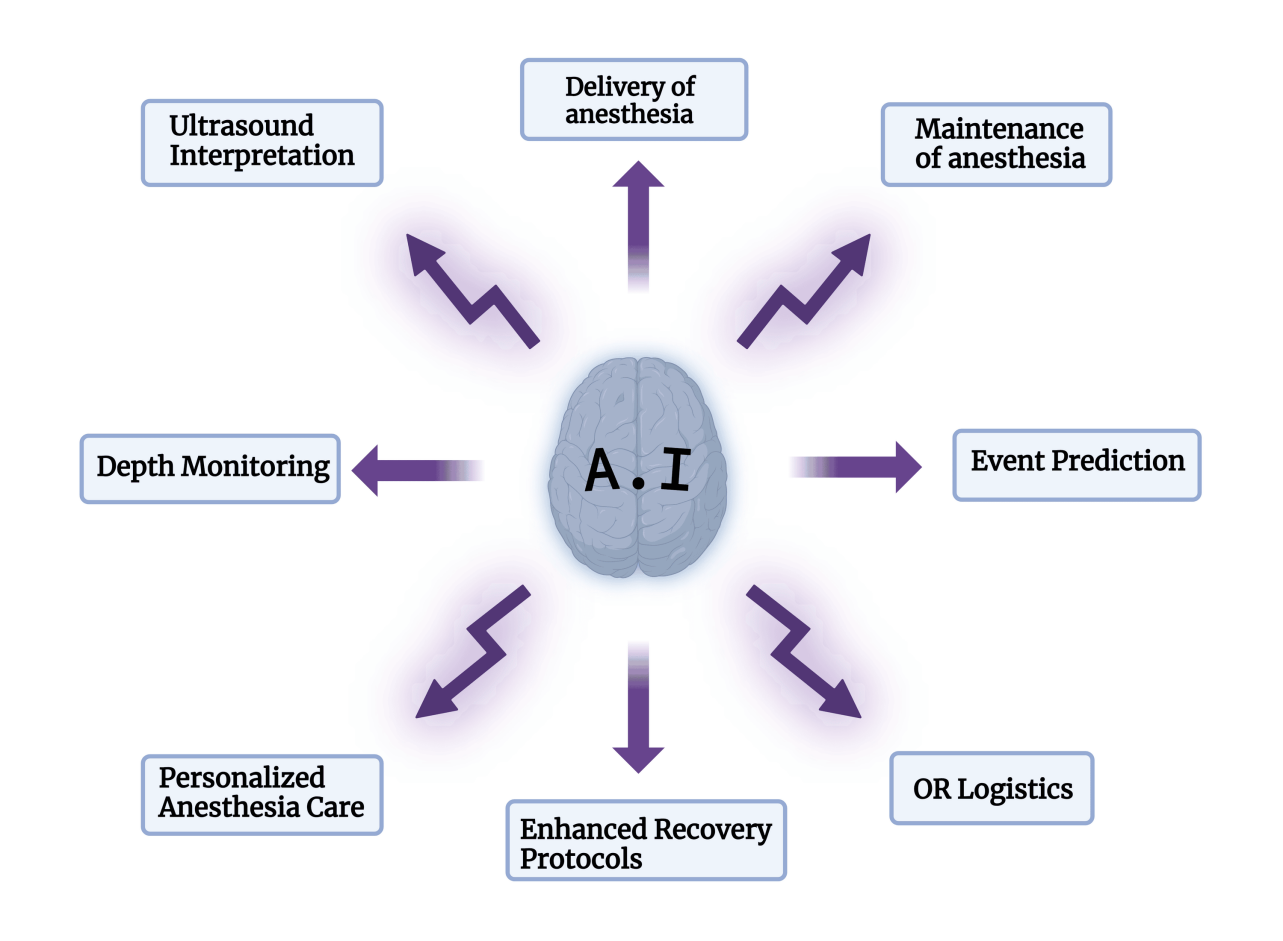
Various applications that will be discussed in this review. OR: operating room Image credits: Arnesh Shukla. Created in Biorender.com [9]

AI has diverse applications in anaesthesiology, including event prediction, depth of anaesthesia monitoring, control of anaesthesia delivery, ultrasound image interpretation, and operating room logistics [7,8]. In this systematic review, we will take a closer look at the current state of AI and do a comprehensive review and discussion of current AI applications in the field of anaesthesiology, summarising existing research and proposing future directions along with its uses and limitations. Figure 1 depicts the various applications that we will discuss in this review.

## Review

Methods

Search Strategy

This systematic review used the Preferred Reporting Items for Systematic Review and Meta-Analysis (PRISMA) 2024 guidelines. To identify relevant literature for our review, we conducted comprehensive searches on several databases, including PubMed, PubMed Central (PMC), PubMed (MeSH), Anesthesiology Journal, Google Scholar, and ScienceDirect. We employed various combinations of keywords, such as "artificial intelligence and anesthesiology" and "machine learning and anesthesiology", among others, to ensure a comprehensive search across all selected databases. Additionally, in PubMed, we implemented a specific search strategy alongside these keywords to further refine our search and identify pertinent literature in the field.

PubMed's MeSH database search is as follows: ("artificial intelligence"(MeSH Terms) OR ("artificial"(All Fields) AND "intelligence"(All Fields)) OR "artificial intelligence"(All Fields)) AND ("anaesthesiology"(All Fields) OR "anesthesiology"(MeSH Terms) OR "anesthesiology"(All Fields) OR "anesthesiology s"(All Fields)). The outcomes of the search strategies are illustrated in Table 1.

**Table 1:** The databases used and the identified numbers of papers for each database.

Search Strategy	Database Used	Number of Papers Identified
Artificial Intelligence AND Anesthesiology	PubMed	2128
Generative Artificial Intelligence AND Anesthesiology	PubMed	626
Generative Artificial Intelligence	Anesthesiology Journal	82
Artificial Intelligence AND Anesthesiology	ScienceDirect	1291

PICO (Population, Intervention, Comparison, Outcome) Criteria

Population: Patients undergoing anaesthesia and perioperative care, as well as anaesthesiologists and healthcare professionals involved in the anaesthesiology practice.

Intervention: Implementation and utilisation of AI in anaesthesiology, including predictive analytics, automated AI tools, and AI-driven monitoring systems.

Comparison: Traditional anaesthesiology practices without the integration of AI devices.

Outcome: Enhanced patient outcomes, improved clinical efficiency, and overall advancement in anaesthesiology practices.

Study design: A comprehensive systematic review and discussion of current AI applications in the field of anaesthesiology, summarising existing research and proposing future directions.

Inclusion and Exclusion Criteria

Our study focus is on "peer-reviewed literature" within the past five years to emphasise the quality of sources. We specifically selected articles written in English or those with available full-text English translations. To ensure relevance to our research topic, we only included studies that involved human participants. Any articles where the full text could not be accessed were excluded from our review. Studies focusing on AI applications outside the field of anaesthesiology were also excluded. We did not include grey literature or proposal papers in our analysis. The inclusion and exclusion criteria are listed in Table 2.

**Table 2:** Inclusion and exclusion criteria. AI: artificial intelligence; ML: machine learning

Inclusion Criteria	Exclusion Criteria
Papers published in the last five years	Grey literature
Papers written in English	Papers focusing on a subset of the population
Human studies	Papers on AI not in the field of anaesthesiology
Papers focusing on ML and the new branches of AI in anaesthesiology	Animal studies

Quality Assessment and Selection Process

The shortlisted articles were imported into EndNote (Clarivate, London, United Kingdom), and duplicate papers were eliminated. Subsequently, each article underwent screening based on titles and abstracts. The remaining articles were then subjected to a thorough evaluation of their full texts, and only those deemed relevant were included in the assessment. Inclusion and exclusion criteria were carefully applied, resulting in the final selection of articles that met the specified criteria.

The shortlisted articles underwent a rigorous quality assessment using appropriate quality appraisal tools. The Newcastle-Ottawa tool was employed to evaluate the quality of observational studies, while the Assessment of Multiple Systematic Reviews (AMSTAR) tool was utilised to assess the quality of systematic reviews. For narrative reviews, the Scale for the Quality Assessment of Narrative Review Articles (SANRA) was applied. Only studies that met the quality appraisal criteria were included in the systematic review, ensuring that only high-quality studies were considered for analysis. The quality appraisal for each study is shown in Table 3, using the appropriate evaluation technique for each type of study.

**Table 3:** Cochrane bias table for quality appraisal. Studies with a high bias in the quality appraisal were excluded from the study selection. D1: Bias from the randomisation process; D2: bias due to deviation from the intended intervention; D3: bias due to missing outcome data; D4: bias in the measurement of the outcome; and D5: bias in the selection of the reported result.

Study ID	D1	D2	D3	D4	D5	Overall
Adams et al. [2]	Medium	Low	Low	Low	Medium	Low
Bellini et al. [3]	Medium	Low	High	Low	Medium	Medium
Hashimoto et al. [4]	Low	Low	Low	Low	Low	Low
Kambale et al. [5]	Low	Low	Low	Low	Medium	Low
Bowness et al. [7]	Low	Low	Low	Low	Medium	Low
Raja et al. [8]	Medium	Low	High	Medium	High	Medium
Chae et al. [10]	Low	Low	Medium	Low	Medium	Low
Tacke et al. [11]	Medium	Low	Low	Low	Low	Low

The study characteristics and their findings are discussed below in Table 4.

**Table 4:** Study characteristics. AI: artificial intelligence; ML: machine learning; DL: deep learning; GAN: generative adversarial networks; BIS: bispectral index; EEG: electroencephalogram; AEP: auditory evoked potentials; RL: reinforcement learning; ANN: artificial neural network; LR: logistic regression; ANFIS: adaptive neuro fuzzy inference system; RMSE: root mean squared error

No.	Author	Year	AI Tool	Key Findings
1	Adams et al. [2]	2023	ML	Leveraging AI to support front-line clinicians can significantly improve patient outcomes but must be used with caution.
2	Bellini et al. [3]	2022	ML	The integration of AI in anesthesia and operating room management shows promise but requires further evaluation before practical implementation due to ethical and societal considerations.
3	Hashimoto et al. [4]	2020	ML, DL	AI has the potential to significantly influence various aspects of clinical anesthesia practice, including perioperative support, event prediction, control of anesthesia delivery, and outpatient management, among other things.
4	Kambale et al. [5]	2024	ML	AI in anesthesia promises to revolutionize patient care by accurately predicting outcomes, optimizing dosing, and providing real-time monitoring during surgery; however, challenges like bias and privacy concerns must be addressed responsibly.
5	Connor et al. [6]	2019	ML	The likely path for integrating AI and ML in anesthesia involves transitioning routine intraoperative patient management to closed-loop control algorithms.
6	Bowness et al. [7]	2022	DL	Non-experts were more likely to provide positive and less likely to provide negative feedback than experts (p=0.001). Positive feedback was provided most frequently by non-experts on the potential role for training (37/60, 61.7%); for experts, it was for its utility in teaching (30/60, 50%). Real-time and remote experts reported a potentially increased risk in 12/254 (4.7%) vs 8/254 (3.1%, p=0.362) scans, respectively.
7	Chae et al. [10]	2020	ML	Supervised ML uses data in matrix form to train models, minimizing prediction errors. Models are evaluated using metrics like area under the curve (AUC), ensuring good performance on both training and validation datasets before real-world deployment.
8	Tacke et al. [11]	2020	ML	A combination of electroencephalogram (EEG) and action evoked potential (AEP) signal parameters developed with automated methods provides a maximum prediction probability of 0.935, which is higher than 0.916 (for EEG parameters) and 0.880 (for AEP parameters) using a cross-validation approach. This suggests that machine learning techniques can successfully be applied to develop an improved combined EEG and AEP parameters to separate consciousness from unconsciousness.
9	Arora et al. [12]	2020	GAN	AI's future ubiquity in clinical practice necessitates evolving anesthesiology training to incorporate AI.
10	Chen et al. [13]	2020	DL	This paper introduces a novel DL framework aimed at improving the accuracy of anesthesia depth prediction by addressing distribution shifts among patients, incorporating historical bispectral index (BIS) values, and suggesting future enhancements such as integrating additional physiological indicators and implementing an attention mechanism to focus on relevant features while also addressing data imbalance.
11	Padmanabhan et al. [14]	2015	RL	Simulation results using 30 patient models with varying pharmacokinetic and pharmacodynamic parameters show that the proposed RL control strategy is promising in designing closed-loop controllers for ICU sedation to regulate sedation and hemodynamic parameters simultaneously.
12	Lin et al. [15]	2002	ANN	In the study, neural networks demonstrated their ability to predict the hypnotic effect of propofol bolus induction. The predictive effects of both neural network models were superior to that of a clinician. The input parameters could not include all the key factors such as cardiac output, central blood volume and some other individual factors, that reduced the predicting power of this model.
13	Lin et al. [16]	2011	ANN	The ANN model had an accuracy of 82.3%, a sensitivity of 76.4%, and a specificity of 85.6%. The accuracy of the LR model was 76.5%, the sensitivity was 74.5%, and the specificity was 77.7%. The area under the receiver operating characteristic curve for the ANN and LR models was 0.893 and 0.840. The clinicians had the lowest predictive accuracy and sensitivity compared with the ANN and LR models.
14	Lin et al. [17]	2008	ANN	The ANN model had a sensitivity of 75.9% and a specificity of 76.0%. The LR model had a sensitivity of 68.1% and a specificity of 73.5%. The area under receiver operating characteristic curves were 0.796 and 0.748. The ANN model performed significantly better than the LR model. The prediction of clinicians had the lowest sensitivity of 28.7%, 22.2%, 21.3%, 16.1%, and 36.1%, and specificity of 76.8%, 84.3%, 83.1%, 87.0%, and 64.0%.
15	Shalbaf et al. [18]	2018	ANFIS	The Adaptive Neuro Fuzzy Inference System being tested classifies EEG data into awake, light, general, and deep states during anesthesia with sevoflurane in 17 patients. Its accuracy is 92% compared to a commercial monitoring system (response entropy index).
16	Mirsadeghi et al. [19]	2016	ML	The experimental results indicate that an overall accuracy of 88.4 % can be obtained using this method for classifying the EEG signal into conscious and unconscious states for all patients. Considering the reliability of this method, we can develop a new EEG monitoring system that could assist anesthesiologists to estimate the depth of anesthesia accurately.
17	Lowery et al. [20]	2013	RL	The results showed that the reinforcement learner compared to a bang-bang controller reduced the dose of the anesthetic agent administered by 9.4% and kept the patient closer to the target state, as measured by RMSE (4.90 compared to 8.47). It also kept the BIS error within a narrow, clinically acceptable range 93.9% of the time. Moreover, the policy was trained using only 50 simulated operations.
18	Zaouter et al. [21]	2016	ML	Robotic anesthesia was successful in 16 patients, which is equivalent to 80% (97.5% confidence interval, 53%–95%) of the patients undergoing cardiac surgery. Four patients were excluded from the final analysis because of technical problems with the automated anesthesia delivery system.
19	Nagata et al. [22]	2023	ML	Automated control achieved via these devices showed non-inferiority compared with anesthesiologists for all three effects of sedation, analgesia, and muscle relaxation. The automated control system also had an improved control accuracy (especially in maintaining adequate muscle relaxation) compared to previous systems.

Results

Study Identification and Selection

**Figure 2:**
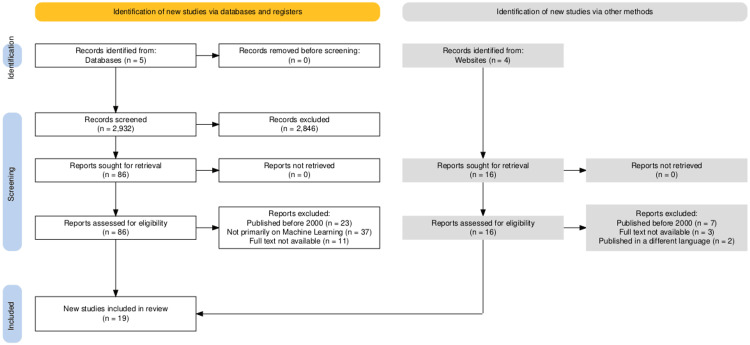
PRISMA flowchart. PRISMA: Preferred Reporting Items for Systematic Reviews and Meta-Analyses [23]

A comprehensive search across all selected databases yielded a total of 4,127 articles. After removing duplicates (904 articles) and irrelevant studies (291 articles) through initial screening based on titles and abstracts, a total of 86 articles remained for further evaluation. The full-text articles from this selection were thoroughly assessed for eligibility and quality, resulting in the final inclusion of 30 articles for the review. Figure 2 presents the detailed selection process in the PRISMA flowchart.

Discussion

With the rapid development of generative AI and machine learning, AI in anaesthesia has become an increasingly popular subject recently, with many articles examining the current developments and the potential advantages and challenges of incorporating AI into anaesthesia practice. Although various branches of AI have been previously described, most of them commonly categorise ML as a significant subfield of AI [4]. Traditional computer programs operate based on explicit instructions to produce specific behaviours from a machine in response to given inputs (e.g., a word processing program's primary function is to display the text input by the user). In contrast, ML enables programs to learn from and adapt to data without explicit programming.

The landscape of ML can be envisioned as fundamentally consisting of three large categories: supervised learning, unsupervised learning, and reinforcement learning [10]. It is necessary to gain a clear overview of the entire field of ML before digging deeper into any specific algorithm. ML refers to a system's ability to acquire and integrate knowledge through large-scale observations and improve and extend itself by learning new knowledge rather than being programmed with that knowledge [24]. The initial step in the ML process is to collect and prepare the data. This involves identifying data sources, gathering them, and then cleaning and preprocessing them. Data cleaning refers to removing irrelevant or incomplete data and ensuring consistency and proper formatting [25]. The next step is exploratory data analysis, which involves analysing the data to identify patterns, correlations, and outliers [25]. The final step before deployment is model selection and training, which depends on the task that must be completed and the characteristics of the data.

**Figure 3:**
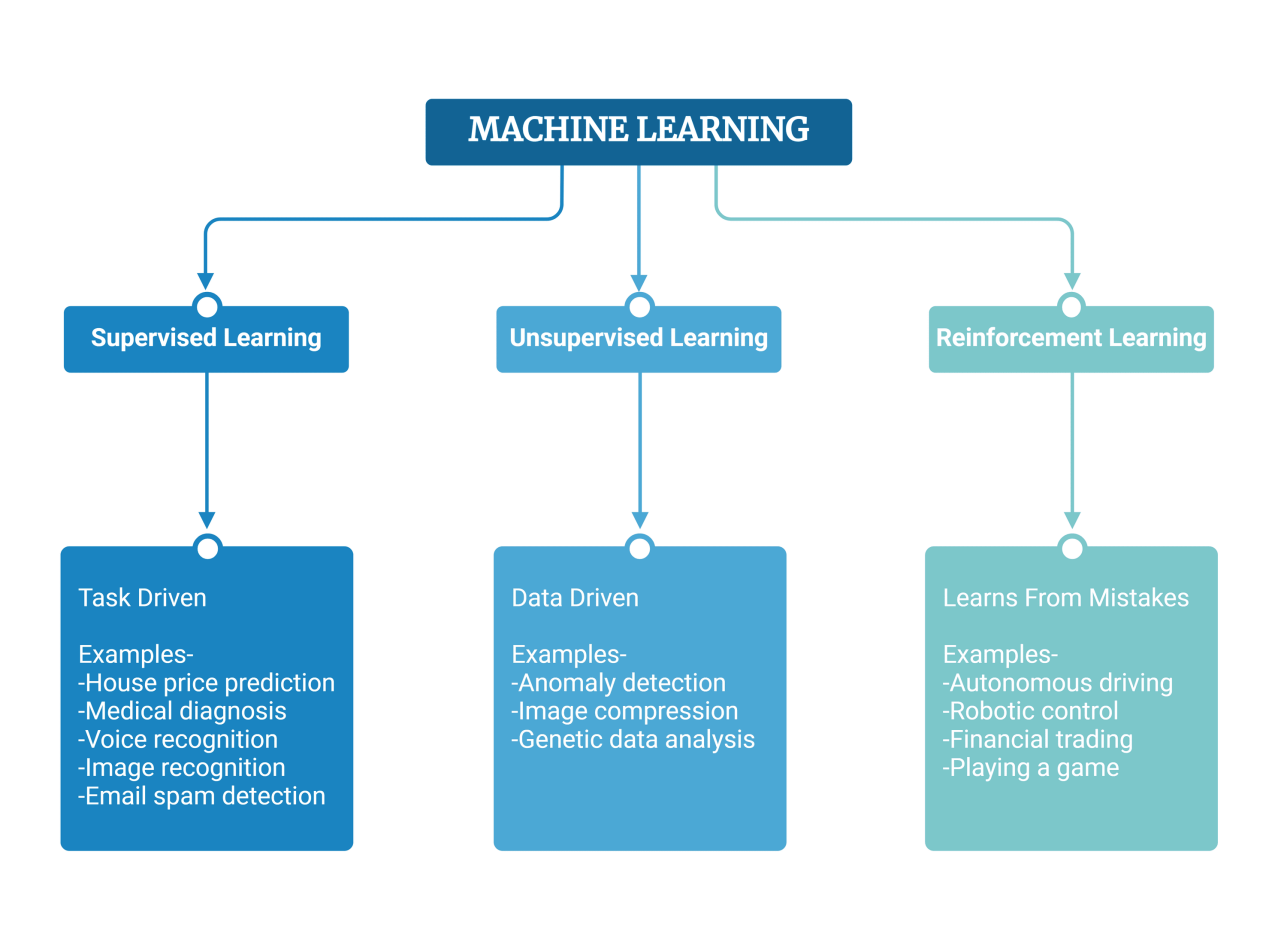
The machine learning (ML) categories and some use-case scenarios for each. Image credits: Arnesh Shukla. Created in Biorender.com [9]

The main classifications of ML models fall under one of three types: supervised learning, unsupervised learning, and reinforcement learning [26]. Figure 3 depicts the ML categories and some use-case scenarios for each.

Supervised learning refers to the ML approach of using labelled data sets. Using labelled inputs, the model can measure its accuracy and learn over time [27]. Unsupervised learning, on the other hand, does not require labelled data sets. Unsupervised learning uses ML algorithms to find hidden patterns in data without the need for human intervention [27]. Reinforcement learning is the third type of ML process by which a system learns to perform a task by trial and error in the absence of any guidance from a human user [28]. Reinforcement learning allows the system to get more accurate and precise with each iteration as it allows the system to learn from its mistakes and improve. Reinforcement learning has played a significant role in advancing the field of AI and has helped integrate AI into clinical practice. An example of this was seen when Padmanabhan et al. performed a study using reinforcement learning to create a closed-loop control of anaesthesia using feedback from the patient's bispectral index (BIS) and mean arterial pressure (MAP) to monitor and control the infusion rates of Propofol in a simulated patient model [14]. Advancements in these different subfields of AI have helped progress the field of AI and ML to new heights. These advancements in generative AI and traditional AI have also led to an increasing number of uses in our healthcare system, specifically in the field of anaesthesiology.

In the field of anaesthesiology, AI has the potential to enhance patient outcomes, reduce healthcare costs, and increase the efficiency of anaesthesia care delivery. Although the application of AI in anaesthesia is still in its early stages, there is a growing interest in exploring its potential uses [5]. In this systematic review, we will look at the different uses of AI in the field of anaesthesiology, along with studies performed on them.

Starting with event prediction, AI has been shown to make highly accurate predictions, surpassing traditional methods in predictive accuracy. A study performed by Lin et al. demonstrated how neural networks could be used to measure the hypnotic effect (as measured by BIS) of an induction bolus dose of Propofol (sensitivity of 82.35%, specificity of 64.38%, and area under the curve of 0.755) [15]. In this study, AI outperformed the average estimate of anaesthesiologists (sensitivity of 20.64%, sensitivity of 92.51%, and area under the curve of 0.5605) in predictive accuracy [15]. The predictive accuracy of AI was also seen in two studies performed by Lin et al. in which artificial neural networks outperformed physicians in predicting post-induction hypotension after general anaesthesia and also outperformed physicians in predicting hypotension after spinal anaesthesia [16,17].

Some of AI's most important potential uses in the field of anaesthesia are automation of anaesthesia delivery and depth of anaesthesia monitoring. Automation of anaesthesia delivery is perhaps the most impactful use of AI and ML in the field of anaesthesiology. To automate anaesthesia delivery, the depth of anaesthesia monitoring plays an important role. Over the years, many different methods of measuring the depth of anaesthesia using AI have been studied and recorded in the literature. ML has been used to monitor many parameters over the years to measure the depth of anaesthesia, including MAP, BIS, and directly using electroencephalographies (EEG). A study performed by Shalbaf et al. used neural networks and fuzzy logic to measure the depth of anaesthesia using EEG signals after administering sevoflurane [18]. This new algorithm was shown to have a 92.91% accuracy compared to the response entropy index, which had an accuracy of 77.5% [18]. This same algorithm was also generalised to measure the depth of anaesthesia in patients sedated with Propofol and sedated with volatile anaesthetics. The algorithm was shown to have an accuracy of 93% compared to BIS, which demonstrated an accuracy of 87% [18]. A study performed by Mirsadeghi et al. showed that ML algorithms analysing various features of direct EEG signals (accuracy of 88.4%) outperformed BIS measurements (accuracy of 84.2%) [19]. Tacke et al. performed a study in which ML used EEG and auditory evoked potential (AEP) parameters to give a maximum prediction probability of 0.935, which was higher than the prediction probability with only EEG signals (0.916) or only AEP parameters(0.880) [11]. This suggests that ML can successfully be applied to develop an improved combined EEG and AEP parameters to measure the depth of anaesthesia [11].

The control of anaesthesia delivery with the help of ML and reinforcement learning has been a topic of growing interest with the advances made in the realm of AI over the past decade. A study by Lowery et al. demonstrated how reinforcement learning algorithms could outperform on-off controllers in the control and delivery of anaesthesia [20]. The study showed that the reinforcement learner, compared to the on-off controller, reduced the amount of anaesthetic administered by 9.4% while keeping the patient closer to the target state as measured by root mean square error (4.90 compared to 8.47) [20]. A clinical trial performed by Zaouter et al. showed very promising results in the feasibility of a completely automated robotic anaesthesia delivery system for cardiac surgery [21]. As the AI industry keeps advancing and improving, robotic anaesthesia will become a reality in the future and will not be far from the realm of possibility. A study by Nagata et al. compared an automated control system using AI for anaesthesia delivery to manual anaesthesia delivery by an anaesthesiologist [22]. The automated control system was able to maintain adequate sedation in 87.21% ± 12.79% of patients, whereas 65.19% ± 20.16% of patients had adequate anaesthesia maintenance in the manual group (p-value < 0.001) [22].

Another use of AI in the field of anaesthesiology is to assist physicians in ultrasound interpretation. A study by Bowness et al. showed how a novel AI device could apply colour overlay on real-time ultrasounds to highlight key anatomical structures [7]. Devices such as these could be beneficial in teaching and training anaesthesiologists. However, despite these promising advances, the implementation of AI in anaesthesiology must be approached with caution, particularly regarding ethical considerations such as data privacy, the potential for algorithmic bias, and the accountability of healthcare providers when AI systems make errors. Continued research, clinical trials, and interdisciplinary collaboration will be essential as AI continues to evolve at a rapid pace.

Limitations

One constraint of our systematic analysis is a small sample size, which may reduce study efficiency. Another potential limitation is that some studies may focus on specific aspects of anaesthesiology or particular patient populations, limiting the generalisability of their findings. The lack of long-term data could be another limitation since ML is relatively new, with most studies focusing on short-term outcomes. In contrast, there is limited data on the long-term outcomes and effects of AI in the field of anaesthesiology. Therefore, to verify the results of this investigation, a prospective, longitudinal study with sufficient power is required in the future.

## Conclusions

AI has the potential to transform anaesthesiology by enhancing patient outcomes, reducing healthcare costs, and improving the efficiency of anaesthesia delivery. It can automate anaesthesia delivery, monitor the depth of anaesthesia, personalise care, and optimise operating room logistics. Many studies show that AI has demonstrated superior predictive accuracy, particularly in managing anaesthetic dosing and predicting patient response. The rapid growth of ML, including supervised learning, unsupervised learning, and reinforcement learning, has played a significant role in these advancements. In particular, DL and reinforcement learning have shown remarkable promise in developing closed-loop anaesthesia systems, which drives progress towards automating anaesthesia delivery. Although the use of AI in anaesthesiology has many benefits, it must be used with caution and consideration. An important consideration is the quality of data used to train AI models. Biased or incomplete data can potentially compromise patient safety, which is why it is crucial to use diverse and comprehensive data sets. Integrating AI in clinical practice raises ethical and legal issues, such as questions regarding accountability if and when an AI system makes an error. Establishing clear guidelines and protocols to address situations such as these is essential. Patient privacy is another major concern, as AI handles large amounts of sensitive patient data.

In conclusion, the incorporation of AI and ML in healthcare, specifically in anaesthesiology, looks very promising and has the potential to revolutionise the field. Continued research focusing on data integrity, algorithm transparency, and ethical frameworks is essential to ensure AI's safe and effective use in anaesthesiology. As AI continues to evolve, its role in anaesthesiology will undoubtedly expand, leading to many opportunities to improve patient care and efficiency in the field of anaesthesiology.
